# An assessment of juvenile Atlantic cod Gadus morhua distribution and growth using diver operated stereo‐video surveys

**DOI:** 10.1111/jfb.12998

**Published:** 2016-05-25

**Authors:** S. A. M. Elliott, P. A. Ahti, M. R. Heath, W. R. Turrell, D. M. Bailey

**Affiliations:** ^1^ Institute of Biodiversity, Animal Health and Comparative MedicineUniversity of GlasgowGlasgowG12 8QQU.K.; ^2^ Department of Mathematics and StatisticsUniversity of Strathclyde16 Richmond StreetGlasgowG1 1XQU.K.; ^3^ Marine Scotland Science, Marine LaboratoryP. O. Box 101, 375 Victoria RoadAberdeenAB11 9DBU.K.

**Keywords:** coastal, habitat, marine protected area, scuba, stereo‐video cameras

## Abstract

Stereo‐video scuba transects were conducted during daylight hours from June to September 2013 within a proposed marine protected area (MPA) in the Firth of Clyde, west of Scotland. More juvenile Atlantic cod Gadus morhua of fork length (L_F_) range 6–11 cm were observed in substrata containing mixed gravel, including maerl, than in boulder‐cobble substrata with high algal cover, or sand with low density seagrass. Community composition was significantly different between substratum types. A decrease in G. morhua abundance was observed over the period of data collection. Over time, mean and variance in G. morhua L_F_ increased, indicating multiple recruitment events. Protecting mixed gravel substrata could be a beneficial management measure to support the survival and recruitment of juvenile G. morhua; other substrata might be important at night given their diel migratory behaviour. Stereo‐video cameras provide a useful non‐destructive fisheries‐independent method to monitor species abundance and length measurements.

## Introduction

With increasing concern over the state of the marine environment, much attention has been paid to the development of marine protected areas (MPAs) as an ecosystem‐based approach to protect vulnerable substrata and restore species and their habitats (Roberts *et al.*, 2005; Seitz *et al.*, 2014). In many cases, however, factors affecting the survival of temperate marine fishes are not well understood (Langton *et al.*, 1996). This is of particular relevance within European waters where measures to restore fish stocks have focused primarily on reducing fishing effort, fishing gear adaptations to reduce by‐catch and fisheries closures (Fernandes & Cook, 2013; Hilborn, 2011). While improvements in some stocks have been observed in the European Union [*e.g*. European anchovy *Engraulis encrasicolus* (L. 1758) and whiting *Merlangius merlangus* (L. 1758)], West of Scotland Atlantic cod *Gadus morhua* L. 1758 stocks remain depleted (Fernandes & Cook, 2013; ICES, 2014).

The Firth of Clyde was once a productive fishery. Commercially important *G. morhua* stocks, however, declined sharply around the 1980s (Thurstan & Roberts, 2010; Heath & Speirs, 2012). Since the first phase of the *G. morhua* recovery plan was introduced (early 2000s) (Anon, 2001; Kraak *et al.*, 2013), measures have been implemented to try and restore stocks, including the prohibition of targeted fishing and a seasonal spawning area closure implemented in the outer Firth of Clyde (Anon, 2001, 2002; Clarke *et al.*, 2015). Today, the predominant fishery occurring in the Firth of Clyde is the Norway lobster *Nephrops norvegicus* fishery, with smaller amounts of scallop dredging and creel fishing occurring (Thurstan & Roberts, 2010; McIntyre *et al.*, 2012). There are various possible reasons for the lack of recovery in *G. morhua* stocks. In the U.K., little attention has been paid to key habitat requirements for juveniles in comparison to Canada, the U.S.A. and Scandinavian countries (Bailey *et al.*, 2011).

To avoid confusion, within the present paper, habitat refers to resources and conditions required by a species to live in during a particular stage of its ontogeny (Hall *et al.*, 1997). Habitat therefore includes the types of substrata (*e.g*. sediment and algae type), physiochemical parameters and biological characteristics required by a species (Gaillard *et al.*, 2010; Elliott *et al.*, 2016). A substratum type is considered important where a change in its conditions or availability has the ability to directly affect the survival of fishes (Langton *et al.*, 1996; Able, 1999). All terminology used in this paper is in line with Elliott *et al.* (2016).

Age‐0 year *G. morhua* are known to migrate into and inhabit shallow (<20 m) nearshore waters between June and October following pelagic larval stages (Magill & Sayer, 2004; Gibb *et al.*, 2007). It is particularly important to understand the habitats of juveniles since cohort size of marine fishes may be determined during their first year (Campana *et al.*, 1989; Myers & Cadigan, 1993; Able, 1999). Juvenile demersal fishes are also thought to occupy a narrower range of substrata than adults (Gibson, 1994; Able, 1999). Higher densities of *G. morhua* have been observed around rocky reefs and eelgrass substrata (Tupper & Boutilier, 1995; Bertelli & Unsworth, 2014), as well as in more exposed areas (Lekve *et al.*, 2006).

Monitoring of fishes in shallow coastal areas containing rocky reefs and boulders is not possible using fisheries‐dependent mechanisms such as demersal trawling gear. Fishing and gear restrictions may also inhibit access in managed areas. Scuba transect methods can be advantageous, reducing damage and mortality to benthos and fishes, and being able to provide greater detail about the association of individual fish with the morphology of the seabed (Gregory & Anderson, 1997). To produce accurate comparative surveys, undertaking standardized diver surveys and minimizing disturbance to fauna can improve precision and reduce bias (Sayer & Poonian, 2007). Stereo‐video cameras are particularly advantageous as they enable accurate measurements to be made (Harvey *et al.*, 2002). Stereo‐video systems have previously been used in tropical and deep sea environments (Cappo *et al.*, 2006; Fitzpatrick *et al.*, 2012) but their application to identify fish substratum association in the U.K. has only recently been trialled through baited camera techniques (Unsworth *et al.*, 2014). Such methods might be a valuable means of collecting information for spatial planning and for monitoring whether management is effective.

The aims of this study were two‐fold: first, to determine the effectiveness of stereo‐video scuba belt transects to assess species abundance and length in U.K. waters and second, to assess abiotic and biotic variables influencing the distribution and abundance of juvenile *G. morhua* in shallow subtidal waters. Data were collected between June and September 2013 around the south of the Isle of Arran, Firth of Clyde. All study sites fell within the South Arran nature conservation MPA (NCMPA) (SNH, 2014), but took place before designation and any new management measures were implemented. By understanding abiotic and biotic variables affecting age‐0 year *G. morhua* abundance and distribution, targeted management measures within the South Arran NCMPA could be implemented to support their survival and apply a more ecosystem‐based management.

## Materials and methods

### Study location

**Figure 1:**
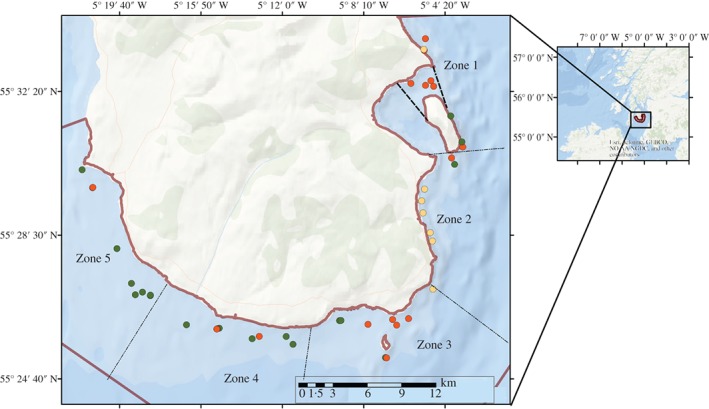
South of Arran nature conservation marine protected area (NCMPA) with dive site locations and substratum categories. 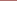, the boundaries of south Arran NCMPA; 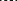, the boundaries of Lamlash Bay no take zone (NTZ); 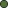, algal‐boulder‐cobble; 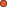, algal‐gravel‐pebble; 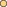, sand.

Data were collected at depths of 4·5–23·0 m around South Arran NCMPA (Fig. 1). South Arran NCMPA encompasses an area of 250 km^2^ and was designated in 2014 for its internationally important seagrass and maerl beds in addition to other substrata (burrowed mud, kelp and seaweed communities) and epibenthic fauna (SNH, 2014). The MPA contains within its boundaries the Lamlash Bay no take zone (NTZ), designated in 2008 and prohibiting all fishing within its boundaries under the Inshore Fishing (Scotland) Act of 1984 (Axelsson *et al.*, 2009).

### Camera set‐up

**Figure 2:**
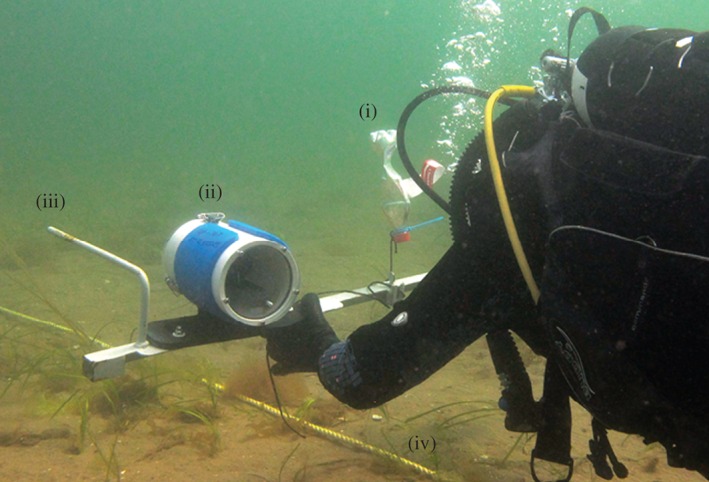
Image of stereo‐video camera and line set‐up showing (i) float for frame buoyancy, (ii) stereo‐video cameras in housing, (iii) custom‐made bar to attach LED lights and (iv) leaded line.

A SeaGIS underwater stereo‐video camera system (SeaGIS, 2013) which consisted of two high‐definition (HF G25, Canon; www.canon.co.uk) video cameras in waterproof housings, attached to a custom‐made diver‐portable steel frame (Fig. 2) was used. The system was set up similar to the prototype described in Harvey & Shortis (1995, 1998); however, this system was optimized for smaller bodied fishes. Distances between cameras were therefore configured with a base separation of 66 cm and an inward calculated angle of view of *c*. 10° in seawater with a visibility of <6 m distance. Each camera was set to manual mode with the focal length set to infinity (∞). Two underwater LED W38VR Archonlight (1400 lumen; www.archonlight.co.uk) torches were mounted on the frame, facing at an angle to the middle of the stereo‐camera field of view. Prior to in‐field data collection, the mounted cameras were calibrated within a controlled environment using methods outlined within Harvey & Shortis (1998) and using the programme and user guide CAL (SeaGIS, 2013). A calibration cube (1 m × 1 m × 0·5 m) containing 85 targets was filmed with the stereo‐video camera system in 20 different orientations (SeaGIS, 2013). Individual camera calibrations were produced using the CAL software and physical camera parameters, camera separation and orientation parameters were computed to allow accurate photographic measurements to be taken (SeaGIS, 2013).

### Data collection

Deployment locations were determined according to existing information collected on substratum types around the pre‐designated MPA (COAST, 2012; SNH, 2014). Stratified random points were identified within five zones (Fig. 1). The zones were created according to prior information on substratum type and wave fetch, using Generate Stratified Random Points with Geospatial Modelling Environment software (Spatial Ecology, 2013) in Arc geographic information system (GIS) version 10.1. These zones were created to provide independent replicates of each substratum type and collect data across a representative range of substrata, depths and wave fetch values. Repeat transects within the same location were not undertaken. Survey work was not conducted in strong tides (measured using tide timetables) and bad weather (heavy rain and wind speed and gusts >15 km h^−1^), because of difficulties in equipment and rigid inflatable boat handling. It has been previously observed that tidal conditions can cause variability in *G. morhua* counts (Sayer & Poonian, 2007).

The abundance of *G. morhua* around south Arran NCMPA was recorded along 100 m strip transects between 5 June and 20 September 2013 (Fig. 1). Since juvenile *G. morhua* had not yet arrived during data collection days in June (5–13), data analysis used 31 transect videos, taken after the first observation of *G. morhua*. Strip transects were chosen as a standard and accurate technique for assessing fish abundance (Kimmel, 1985; Hunter & Sayer, 2009). A leaded line transect was laid perpendicular to the shore line to keep survey depth consistent within the transect. Following a 10 min wait for any disturbance to the seabed or fauna to dissipate (Dickens *et al.*, 2011), the divers descended and swam at a slow constant speed along the transect. Transects were carried out by scuba divers swimming *c*. 1 m above the seabed with cameras held at a slight downward angle to capture fauna in front of the field of view and the substratum. An index of maximum horizontal visibility was measured using a Secchi disc attached to the end of the leaded line. The maximum distance at which it could be distinguished was measured in the stereo‐video recordings. An LED diode was used to synchronize the video footage prior to surveys and following transect completion (Harvey & Shortis, 1995). To reduce diel effects on species, data collection took place between 0800 and 1500 hours (GMT), a minimum of 3 h after sunrise and before sunset. As a result of logistical complications, night sampling did not take place.

### Video analysis

Each transect video was analysed twice by two observers using Event Measure software (SeaGIS, 2013) to reduce observer bias. The first analysis focused on substratum characterization, the second on fauna identification, abundance and length measurements. In the absence of acoustically mapped substrata around south Arran, substratum categories were visually classified according to the most abundant combination of sediment grain sizes and macrophyte types observed together (Table I), similar to Gregory & Anderson (1997) and Cote *et al.* (2001, 2003). As transects had a uniform combination of sediment and algae type, transects were assigned a single overall transect substratum type using the two most common divisions on the Wentworth scale sediment (Wentworth, 1922; Connor *et al.*, 2004) and broad algae type and density (estimated by percentage cover; Table I). Seagrass was not treated separately to sand because of the low density and spatial extent within the area, and the small sample size of the dataset. Equally, maerl was not treated separately from gravel‐pebble substratum type because of its gravel‐pebble sized form around south of Arran. In addition impacted maerl has been demonstrated to be more similar to gravel than live maerl (Kamenos *et al.* 2003). As a result of insufficient prior knowledge of the substratum types of the area, the experimental design was unbalanced. Fourteen algal‐boulder‐cobble substratum type transects were carried out compared with 12 algal‐gravel‐pebble transects and five for the sand substratum category.

**Table I:** Substratum type characterized according to dominant sediment type and macrophyte type and density

Substratum type	Sediment composition	Algae and seagrass type and density
Algal‐boulder‐cobble (ABC)	Sediments composed of mixed boulders and cobbles (particles > 6·4 cm)	Sediment covered in a mixture of kelp and red algae (>60% algae cover). Examples of algae species include *Laminaria* spp. and *Ceramium* spp.
Algal‐gravel‐pebble (AGP)	Mixed gravel (stone, shell and maerl), *Phymatolithon calcareum* and pebble (particles 0·4–6·4 cm)	Between 20 and 50% of sediment covered by algae
Sand	Sandy sediments which may contain some gravel (consisting of broken shell) (particles < 0·4 cm)	<25% algae or seagrass *Zostera marina* cover

Sections of the video recordings where the camera angle was incorrect and the substratum was not visible were removed and the transect length was adjusted in subsequent calculations. Any further distance lost from transect length caused by large boulders or slack line was deducted from the total length of the transect. One entire transect was removed from the analysis because of inappropriate field of view. For each transect, the width of the field of view of the video camera was measured by identifying recognizable points on the seabed on both cameras. Horizontal visibility along the transect was measured in the video recordings as the greatest distance at which the Secchi disc was visible.

Fauna were identified to the lowest taxonomic level possible, usually to species. The fish fork length (*L*_F_) measurements were taken (measuring from the nose to the fork). To undertake *L*_F_ measurements, each individual observed had to be visible in both cameras. *L*_F_ measurements of all *G. morhua* observed were therefore not possible. All *L*_F_ measurements with a root mean square (RMS) error above 2 cm and with a precision of *L*_F_ measurement >0·5 cm were removed from the analysis (SeaGIS, 2013).

### Data analysis

To understand community composition differences between substratum types, permutation analysis of variance (PERMANOVA) in PERMANOVA 6 software as described in Anderson *et al.* (2008) was undertaken. PERMANOVA was used in order to overcome distributional and homoscedasticity restrictions of ANOVA. The standardized abundance of species was square root transformed to reduce the influence of dominant species. A Bray–Curtis similarity coefficient was used prior to applying PERMANOVA. Posterior pair‐wise tests were used to compare the difference between the groups of samples. The PERMANOVA was run with 9999 permutations to draw inferences at the *P*_(perm)_ < 0·001 level. Visualization of the matrices was achieved using non‐metric multi‐dimensional scaling (nMDS) plots which provide values of stress (stress increases with reduced dimensionality or the ordination). Similarity percentages (SIMPER) analysis was used to determine which species contributed most to the dissimilarity between the different substratum types (Clarke & Warwick, 2001).

The effect of abiotic habitat variables on age‐0 year *G. morhua* abundance included: substratum type, depth (m), distance from coast (m), Julian date (*J*_D_, days) and wave fetch (km). Wave fetch values for a 200 m coastline grid (downloaded from www.sams.ac.uk/michael‐burrows) were used as described in Burrows *et al.* (2008). For each transect location, wave fetch for the closest grid was obtained. Distance from coast was calculated using Arc GIS version 10.1. Biotic variables explored included: Hill diversity *N*_2_ (reciprocal of Simpson's index) and *N*_∞_ (reciprocal of the proportional abundance of the commonest species) (Hill, 1973) for epibenthic fauna (*e.g*. tunicates, echinoderms and crustaceans). Difficult to identify fauna, *e.g*. hydroid, bryozoan and *Majidae* spp., could not always be identified to species level. For continuity of analysis, such fauna were quantified in total visible hydroid and bryozoan or *Majidae* abundance (Unsworth *et al.*, 2014).

To condense multivariate variability into fewer dimensions and identify habitat variables affecting the distribution of *G. morhua*, a principal component analysis (PCA) was performed using R software (version 3.03; R Core Team; www.r‐project.org). Explanatory variables observed to have a stronger effect on *G. morhua* abundance from the PCA were used in a generalized linear model (GLM) to understand *G. morhua* abundance, removing collinear variables. An offset for transect area (m^2^) was incorporated into the GLM. A negative binomial distribution was used to account for over dispersion. Explanatory variables included substratum type (three levels), Hill diversity index for epibenthic fauna (continuous), wave fetch (continuous) and *J*_D_ (treated as a continuous variable to reduce the number of parameters used in the model). The model of best fit was log *Y*_*i*_ = *β*_0_ + *β*_1_, *S*_*ij*_ + *β*_2_, *J*_D*i*_ + offset(transect area)_*i*_, where *Y_i_* is *G. morhua* abundance, *β* the coefficient, , *S_ij_* substratum type and, *J*_D*i*_ is the Julian date. A random effect for zone using R package ‘glmmADMD’ (Skaug *et al.*, 2014) was tested for but was not significant. Tukey tests using R package ‘multcomp’ (Hothorn *et al.*, 2008) were used to test the difference between categorical variables. Backwards stepwise model selection was implemented (Bolker *et al.*, 2009; Zuur *et al.*, 2009) and a log likelihood ratio test was used to test model significance against the null hypothesis in addition to checking residual plots.

A general linear mixed model (GLMM) using R package ‘nlme’ (Pinheiro *et al.*, 2014) was used to model length measurements. The best model fit included *J*_D_ as a fixed effect with an offset for the transect area, and a random effect for zone: *Y*_*i*_ = *β*_0_ + *β*_1_, *J*_D*i*_ + offset(transect area)_*i*_ + *b*_*ij*_, where *Y_i_* is *G. morhua L*_F_, *β* the coefficient are the coefficients, , *J*_D*i*_ the Julian date and , *b_ij_* is the random effect for zone. A large outlier identified by Cleveland dotplot was removed from analysis since it was considered that the individual could have been of age 1 year.

## Results

Thirty‐one stereo‐video scuba transects were analysed, covering an area of 4093·14 m^2^ (mean ± s.d. transect length = 95·56 ± 10·23 m and mean ± s.d. transect width = 1·38 ± 0·18 m) (Fig. 1). A total of 496 *G. morhua* were identified with a mean ± s.d. of 11·41 ± 19·47 per transect and within four of the 31 (13%) transects no *G. morhua* were observed. Forty‐five taxonomic groups were identified from 34 different families. Ninety per cent (9327) of the fauna identified were classed as epibenthic fauna. The maximum distance *G. morhua* were able to be identified and measured accurately was 2·86 m from the cameras (mean ± s.d. = 1·52 ± 0·39 m) and the minimum distance objects were measured was 0·85 m. The maximum distance the Secchi disc was seen from the cameras varied between 4 and 5·5 m. It is therefore unlikely that varying underwater visibility affected identification and measurement analysis.

**Figure 3:**
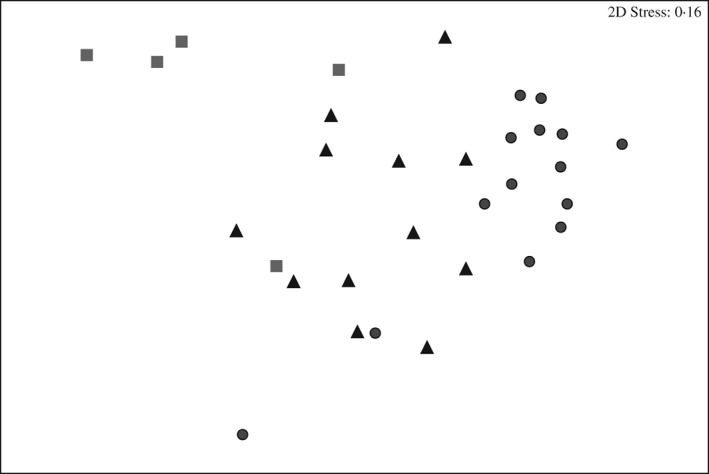
nMDS plot of the community composition of all fauna observed between substratum types (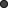, algal‐boulder‐cobble; 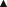, algal‐gravel‐pebble; 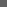, sand). Significant effects of substratum type on assemblage structure are observed (PERMANOVA, P < 0·001).

Differences in community composition between substratum types were observed (pseudo‐*F* = 2·33, *P*_(perm)_ < 0·001). Pair‐wise tests between substratum type showed significant differences between algal‐gravel‐pebble (AGP) and algal‐boulder‐cobble (ABC) (*t* = 1·63, *P*_(perm)_ < 0·001) and ABC and sand substratum type (*t* = 1·99, *P*_(perm)_ < 0·001). No significant difference between AGP and sand substratum type was observed (*t* = 0·91, *P*_(perm)_ > 0·05). The nMDS plot (Fig. 3) shows relatively good ordination (stress 0·16), with some overlap between substratum types. SIMPER analysis showed 22 species were required to explain dissimilarity between substratum types with 80% dissimilarity between AGP and sand, 79% between AGP and ABC and 94% between ABC and sand. Hydroids and poor cod *Trisopterus minutus* (L. 1758) featured in the top species causing the largest dissimilarity between AGP and sand and AGP and ABC. Burrowing anemones *Ceriantheopsis lloydii* and the common sea urchins *Echinus esculentus* led to greatest dissimilarity between ABC and sand (cumulative dissimilarity of 19%).

### Abiotic and biotic effects on G. morhua abundance

The PCA was conducted on seven variables. Two components had eigenvalues over Kaiser's (1960) criterion of 1, and in combination explained 57% (PC1 35%, PC2 22%) of the variance. PC1 was most negatively correlated with Hill diversity indices followed by substratum type and positively correlated with wave fetch_._ PC2 correlated most strongly with distance from coast with a negative correlation with substratum type (Table II). These results indicate that *N*_2_, substratum type, distance from coast and wave fetch had stronger trends than other variables and were therefore used as explanatory variables to understand the abundance and distribution of *G. morhua*.

**Table II:** Eigenvectors of the standardized first and second principal components from the PCA of seven Gadus morhua habitat variables

Variable	PC1	PC2
Depth	0·240	0·268
Distance from coast	0·258	0·568
*J*_D_	−0·175	0·265
*N*_2_	−0·539	0·360
*N*_∞_	−0·512	0·410
Substratum type	−0·448	−0·261
Wave fetch	0·301	0·409

*J*_D_, Julian date; *N*_2_, Hill diversity *N*_2_ (reciprocal of Simpson's index); *N*_∞_, reciprocal of the proportional abundance of the commonest species.

**Figure 4:**
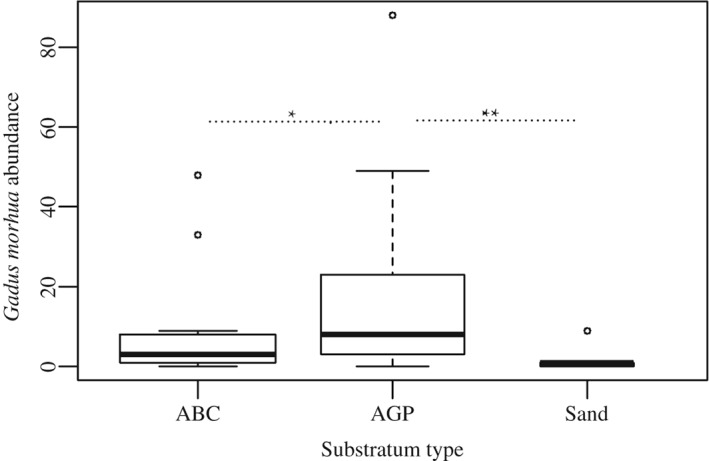
Substratum type association of age‐0 year group Gadus morhua observed around south Arran nature conservation marine protected area (NCMPA). More juveniles were found in relation to substratum type algal‐gravel‐pebble than algal‐boulder‐cobble or sand. No significant difference was observed between algal‐boulder‐cobble and sand. The varied width boxplots, proportional to the square root of the sample sizes, indicate the 25th and 75th percentiles of the total number of G. morhua observed within the different substrata. The upper bars indicate the 10th and the lower bars the 90th percentiles. The 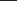 indicates the median size. 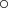 indicate the outliers. 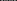 between substratum types with * refers to Tukey test P‐value significance (*, P < 0·05; **, P < 0·01).

**Figure 5:**
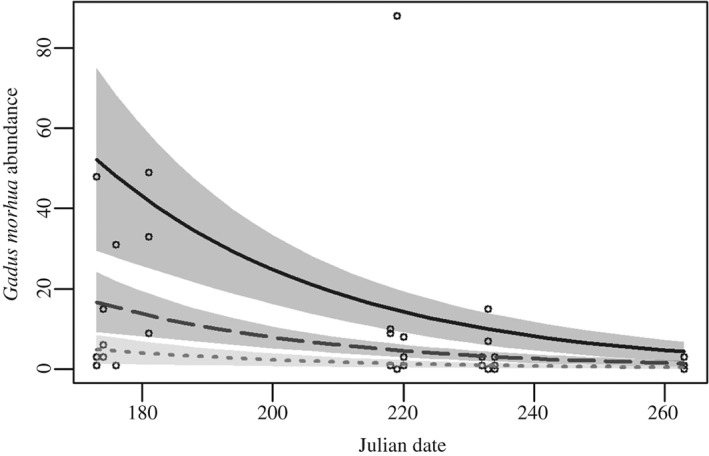
Gadus morhua abundance for each substratum type over the course of data collection. 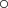, abundance of G. morhua from 22 July to 20 September 2013. 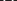, algal‐boulder‐cobble; 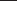, algal‐gravel‐pebble; 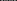, sand GLM fitted lines; shaded area indicates ±95% c.i. A decline in G. morhua abundance was observed over the course of data collection (P < 0·01).

Analysis of the explanatory variables independently, only substratum type and *J*_D_ had an effect on the abundance of juvenile *G. morhua* [*L* = 95·32 (d.f. = 5, theta = 0·48, *P* < 0·01)]. The highest abundance of juvenile *G. morhua* was observed within AGP substratum type, and the lowest abundance was observed in sand substratum type. Intermediate values were observed in ABC (Fig. 4 and Appendices I and II). A decrease in the abundance of *G. morhua* was observed over the period of data collection (Fig. 5 and Appendix I).

### Length analysis

**Figure 6:**
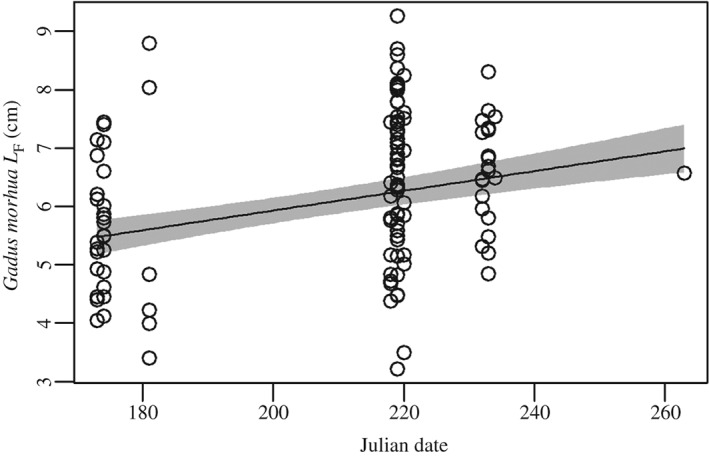
Gadus morhua fork length (L_F_) over the course of data collection. Data points represent L_F_ of G. morhua measured from 22 July to 20 September 2013. 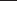, the GLMM fitted line; 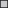, ±95% c.i. An increase in age‐0 year G. morhua L_F_ was observed over the course of data collection (P < 0·01).

One hundred and twenty‐one *G. morhua L*_F_ measurements were made with a mean ± s.d. of 6·3 ± 1·4 cm. The largest *G. morhua* observed was 11·4 cm and the smallest 3·2 cm. The largest individual (2 cm larger than the second largest individual) was excluded from analysis as it could have been a small age 1 year individual following exploration of Marine Scotland Science quarter three (July to September) scientific bottom trawl data. All other *G. morhua* analysed were deemed to be age 0 year (Dalley & Anderson, 1997; Marty *et al.*, 2014). An increase in *L*_F_ was observed over the course of data collection [*L* = −470·50 (d.f. = 4, *P* < 0·01); Fig. 6 and Appendix III]. No other variables were significant in explaining *G. morhua L*_F_. An increase in *L*_F_ variation was also observed over this time period (LM, *F*_1,118_ = 9·18, *P* < 0·01) [*L* = −547·30 (d.f. = 3, *P* < 0·01)] (Appendix IV).

## Discussion

As far as is known, this is the first study using stereo‐video scuba transects in the North Atlantic Ocean and builds upon existing single camera and underwater visual census (UVC) studies (Schneider *et al.*, 2008; Hunter & Sayer, 2009). Stereo‐video scuba transects permit accurate, fisheries‐independent, three‐dimensional measurements of fauna and transect dimensions to be made (Harvey *et al.*, 2002). Data collected have enabled fine‐scale abundance and distribution information to be gathered for the first time on *G. morhua* during daylight hours within the Firth of Clyde.

The abundance of juvenile *G. morhua* varied with substratum type, with more *G. morhua* observed in algal‐gravel‐pebble substrata than algal‐boulder‐cobble or sand. Juvenile *G. morhua* exhibit a light brown and white checkerboard pattern which on gravel‐pebble surfaces makes them relatively difficult to distinguish from their background, obscuring their movement from predators (Gregory & Anderson, 1997). The combination of colouration and substrata of sufficient rugosity to seek refuge within suggests that age‐0 year *G. morhua*, of the size ranges observed, may choose to spend a greater proportion of their time on algal‐gravel‐pebble substratum type. Similarly, Lough *et al.* (1989) observed juvenile *G. morhua* in high abundance on pebble‐gravel substrata. The high variability associated with these observations (Fig. 4) is likely to be a consequence of the small sample size and some variability in juvenile *G. morhua* substratum selection.

Transects within Lamlash Bay NTZ were algal‐gravel‐pebble substratum types. The effect of the NTZ on juvenile *G. morhua* abundance was not explored as data on juvenile gadoid abundance were not available prior to its establishment to perform a before‐after control impact study (Sale *et al.*, 2005). A study undertaken by Howarth *et al.* (2015) found no difference in fish abundance within and out‐with Lamlash Bay NTZ. The latter may be a result of the reserve's small size (2·67 km^2^) and its young age (Howarth *et al.*, 2015). Previous research on juvenile *G. morhua* does, however, show limited movement (Grant & Brown, 1998) but this may vary depending on substratum type (Laurel *et al.*, 2004).

Seagrass beds have previously been observed to be nursery grounds for age‐0 year *G. morhua* (Linehan *et al.*, 2001; Bertelli & Unsworth, 2014; Lilley & Unsworth, 2014) with some studies showing increased nocturnal association (Anderson *et al.*, 2007; Bertelli & Unsworth, 2014). Because of the sample size and low density of *Z. marina* sampled within the area, this substratum was merged with sand. Low‐density seagrass areas have been related to be more similar to sandy sites (Jackson *et al.*, 2001; McCloskey & Unsworth, 2015), particularly when patchy with low shoot density and area coverage (Jackson *et al.*, 2001; Gorman *et al.*, 2009). Mixed diurnal behaviour has also been observed with age‐0 year *G. morhua*, with some experiments showing more active behaviour during daylight hours (Keats & Steele, 1992; Sayer & Poonian, 2007). Differential aggregation behaviour has also been observed depending on light levels, predator presence and seagrass patch size (Laurel *et al.*, 2003, 2004; Anderson *et al.*, 2007).

Gotceitas & Brown (1993) observed that juvenile *G. morhua* within an experimental tank selected cobble substrata in the presence of predators whilst selecting sand and gravel‐pebble substrata in the absence of predators. It is possible that the juveniles identified during data collection did not feel threatened by the diver, and the low abundance of larger piscivores (Heath & Speirs, 2012) may have led to higher abundances on algal‐gravel‐pebble substratum type. In this study, no predator–prey interactions were observed. It is thought that some gravel substrata, specifically containing maerl, may contribute to higher species diversity, structural rugosity (relative to the size of *G. morhua*) and heterogeneity, and that these factors are of importance to the survival of juvenile *G. morhua* (Hall‐Spencer *et al.*, 2003; Kamenos, 2004; Lough, 2010).

A decline in *G. morhua* abundance and an increase in juvenile size and size variation were detected over the course of data collection. *Gadus morhua* have been observed to arrive in recruitment pulses to coastal areas during downwelling events (Ings *et al.*, 2008). The increase in size variation is most likely caused by pulse recruitment occurring over this time period, or one continued long pulse recruitment (Bastrikin *et al.*, 2014) from July to August 2013. The decline in abundance is unlikely to have been caused by fish moving into deeper waters within such a narrow time span since previous studies show that this migration occurs after their first winter or first year (Magill & Sayer, 2004).

Fewer *L*_F_ measurements than counts were made (24% of the total number of *G. morhua*) owing to a combination of not being able to distinguish individual juveniles within schools in both cameras and a blind spot between the cameras where the *G. morhua* were too close to the cameras to be measured (Unsworth *et al.*, 2014). This latter problem could have been reduced by having the cameras closer together, but at the expense of reduced accuracy at distance (Boutros *et al.*, 2015). Precision in the Z direction (towards and away from camera) is affected by the distance between cameras, affecting all measurements of objects which are not angled normal to the camera axis (SeaGIS, 2013; Boutros *et al.*, 2015).

Future temperate water studies should take water visibility and fish size into account in order to maximize the number of fish measured. Stereo‐video scuba transects can provide detailed and valuable information on fish assemblage and population structure in rocky and sensitive substrata which would otherwise be inaccessible. Use of semi‐closed or closed circuit rebreather apparatus, or autonomous underwater vehicles (AUV) may further reduce observer bias (Sayer & Poonian, 2007; Clarke *et al.*, 2009). With the rise in MPAs and spatial restrictions to manage substrata and species around the U.K., this technique provides important information for fisheries management and information for possible future monitoring.

Despite measures in place to recover stocks, the already low numbers of *G. morhua*, small length index and isolation of the Firth of Clyde in comparison to neighbouring areas are likely to cause it to be more susceptible to local fishing impact (Heath & Speirs, 2012). Much debate exists on the value of MPAs for the protection of fishes, particularly in temperate environments (Roberts *et al.*, 2005; Takashina & Mougi, 2014; Fernández‐Chacón *et al.*, 2015). If an MPA can protect important substrata of value to juvenile *G. morhua*, bottle neck recruitment may be avoided, thus increasing the survival of individuals at this critical stage in their life cycle (Lough, 2010). Management measures have recently (December 2015) been established to recover maerl beds found within the NCMPA (Scottish Government, 2015). On the basis of the data presented here, it appears that such management measures could have benefits for juvenile *G. morhua*. In the meantime, further investigations are recommended to strengthen habitat‐related observations of juvenile *G. morhua* abundance and distribution. Better understanding and protection of important habitat components could support juvenile *G. morhua* survival and recruitment.

The authors would like to thank the two independent reviewers for their helpful feedback on this manuscript. Thanks to J. Clarke, C. Willmott, C. Hopkins and D. McNeil for dive technical assistance and H. Wood and R. Cheshire for the boat handling expertise and use of their RIBs to collect data. We also thank H. Wood and Scottish Natural Heritage for information and data on benthic substrata within the area around south Arran, and H. Wood for the many other types of logistical support he provided. Thanks to P. Johnson for statistical advice. Finally, the first author would like to give a special thanks to Marine Scotland (Clyde 2020) and the ClimateXChange centre student support and NERC National facility for Scientific Diving (Grant NFSD/13/01) for scientific diving training without which collection of the data would not have been possible.

## Appendix

**Table:** APPENDIX I. Results from the model of best fit for the response variable Gadus morhua abundance. Explanatory variables show substratum type and Julian date (J_D_) with an offset of transect area (m^2^). Coefficients and diagnostics (Z‐ and P‐values) indicate the effect of each parameter level on the reference level, denoted as intercept. The reference level is substratum type, algal‐boulder‐cobble (ABC)

Variables	Estimate	s.e.	*Z*‐value	*P*‐value
Intercept	3·1028	1·6938	1·8320	>0·05
AGP	1·1524	0·4721	2·4410	<0·05
Sand	−1·2494	0·7448	−1·6780	>0·05
*J*_D_	−0·0280	0·0079	−3·5370	<0·001

AGP, algal‐gravel‐pebble.

**Table:** APPENDIX II. Results from the Tukey test performed between substratum type categories for the response variable Gadus morhua abundance

Variables	Estimate	s.e.	*Z*‐value	*P*‐value
AGP‐ABC	1·1386	0·4757	2·393	<0·05
Sand‐ABC	−1·2227	0·7547	−1·620	>0·05
Sand‐AGP	−2·3613	0·7595	−3·109	<0·01

AGP, algal‐gravel‐pebble; ABC, algal‐boulder‐cobble.

**Table:** APPENDIX III. Results from the model of best fit for the response variable Gadus morhua fork length (L_F_). Fixed effects show Julian day (J_D_) with an offset of transect area (m^2^)

Variables	Estimate	s.e.	*t*‐value	*P*‐value
Intercept	23·30671	12·51339	1·862542	>0·05
*J*_D_	0·182449	0·059287	3·077398	<0·01

**Table:** APPENDIX IV. Results from the model of best fit for the response variable Gadus morhua fork length (L_F_) variation over the period of data collection

Variables	Estimate	s.e.	*t*‐value	*P*‐value
Intercept	−9·6035	20·48846	−0·469	>0·05
*J*_D_	0·2948	0·09732	3·030	<0·01

*J*_D_, Julian day.
